# Body mass index in relation to prostate-specific antigen-related parameters

**DOI:** 10.1186/s12894-020-00746-8

**Published:** 2021-09-16

**Authors:** Dandan Lin, Ting Liu, Luling Chen, Zongtao Chen

**Affiliations:** Health Management Centre, The First Affiliated Hospital of Army Medical University, Chongqing, China

**Keywords:** Body mass index, Prostate-specific antigen, Prostate-specific antigen density, Prostate-specific antigen mass

## Abstract

**Background:**

Only a few previous studies conducted to assess the association between body mass index (BMI) and prostate-specific antigen (PSA) related parameters have taken prostate volume (PV) and blood volume (BV) into consideration. The objective of this study was to assess the relationship between BMI and parameters of PSA concentrations in Chinese adult men.

**Methods:**

A total of 86,912 men who have received annual physical examination at the First Affiliated Hospital of Army Medical University from 1 January 2011 to 31 December 2018 were included in this study. Linear regression models were performed to assess the relationship between BMI, PV, BV and PSA, and analyze the correlation between BMI and PSA, PSA density and PSA mass.

**Results:**

The univariable linear regression showed that PV, BV, systolic pressure (SBP), pulse, fasting blood glucose (FBG) and age were significantly associated with PSA level (*P* < 0.05). The multivariate linear regression demonstrated that PV, BV, FBG and age were significantly associated with PSA level (*P* < 0.05). WHR and BMI is negatively associated with PSA and PSA density (*P* < 0.05), and no statistically significant association was found between PSA mass and WHR and (*P* = 0.268) or BMI (*P* = 0.608).

**Conclusions:**

The findings of this large-sample, hospital-based study in China indicate that PV was positively associated with serum PSA concentrations, while BMI and BV were inversely related with PSA levels. PSA mass can be used to estimate the PSA concentration without being affected by obesity in Chinese men.

## Background

Prostate cancer is a leading cause of death among men in the developed countries [1]. In 2012, 1.1 million men were diagnosed with prostate cancer worldwide, accounting for 15% of all cancers diagnosed in men according to the World Health Organization's International Agency for Research on Cancer [2]. However, screening for prostate cancer was one of the most hotly debated health care issues due to its controversy, overtreatment, psychological distress, and unnecessary medical cost [3].

Prostate specific antigen (PSA) is the most common predictor for early screening and diagnosis of prostate cancer although there are still some challenges with PSA test [4]. A relationship between obesity and low PSA levels has been identified in several studies [5–8]. Obesity plays a key role in developing abnormalities in sex hormone metabolism and insulin levels, because of the excessive accumulation of adipose tissue or body fat. However, the specificity of PSA is low and the false positive rate is relatively high, as most men who undertake biopsy for elevated PSA levels are not diagnosed with prostate cancer [9]. The most accepted hypothesis was that the men with a higher BMI might have enlarged prostate [10, 11] and blood volume (prostate volume and blood volume) [8, 12], which could lead to the underestimation or overestimation of serum PSA levels.

However, few large-scale studies in China conducted to assess the association between BMI and screening and diagnosis parameters of prostate cancer have taken prostate volume (PV) and blood volume (BV) into consideration. The aim of this study is to assess the relationship between BMI, PV, BV and PSA in Chinese men, and whether there is a PSA related parameter that is not affected by BMI and could be used for the diagnosis of prostate cancer based on the data collected in physical examination of the residents of Southwest China.

## Methods

### Overall study design

From 1 January 2011 to 31 December 2018, 86,912 consecutive ostensibly healthy Chinese men received physical examination in the Health Management Department of the First Affiliated Hospital of Army Military Medical University. The PSA measures were collected as part of early screening of prostate cancer of these men. The criteria for inclusion in this study were as follows: physical examination, PSA testing, and prostate ultrasound testing were performed; there were no obvious abnormalities in prostate ultrasound diagnosis; there was no history of prostate cancer and prostate surgery.

### Clinical variables

The information of physical examination of the participants was collected, including age (year), height (cm), weight (kg), PSA level (ng/ml) and prostate volume (ml). BMI (kg/m^2^) was defined as weight (kg) divided by the square of height (m^2^). According to WHO’s BMI grading standards for the Asia–Pacific region, the recruited subjects were divided into: normal weight (18.5 kg/m–23.9 kg/m^2^), overweight (24 kg/m^2^–27.9 kg/m^2^) and obese (BMI > 28 kg/m^2^). Body surface area (m^2^) = weight ^0.425^ × height ^0.725^ × 0.2025. BV (L) = body surface area × 1.67. PV (ml) = left and right diameter × front and back diameter × up and down diameter × 0.52. PSA density (μg) = PSA/PV. PSA mass = PSA × BV. A blood sample was obtained for serum PSA. All anthropometric measurements were made by trained observers using standardized techniques. All measures collected in this retrospective study were part of the routine physical examination.

### Statistical analysis

After a log transformation, the values of PSA, PSA density, and PSA mass were normally distributed, and then a linear regression was performed to quantify the relationship between BMI and PSA parameters. The SPSS 20.0 software (SPSS, Inc, Chicago, IL) was used for statistical analysis and *P* < 0.05 was considered significant for all analysis.

## Results

### Baseline characteristics of participants

In the present study, all data collected from 86,912 men were analyzed (age range: 18–98, with the median age of 46). The median BMI was 24.88 kg/m^2^, the median PSA level was 0.45 ng/mL, the median PV was 16.97 mL, the median PSA density level was 0.026 μg, the median BV level was 2.96 L, and the median PSA mass level was 1.34 μg. The minimum and maximum values of these variables were also presented. The demographics of the study population are listed in Table 1.

**Table 1:** Characteristics of the study population

Factors	Median	P_25_	P_75_	Min	Max
Age (years)	46	38	53	18	98
Height (cm)	166.5	162.5	170.8	134	202
Weight (kg)	69	62.9	75.5	39.6	126.7
BMI (kg/m^2^)	24.88	22.93	26.89	18.50	43.37
PSA (ng/ml)	0.45	0.27	0.76	0.01	2.99
PV (ml)	16.97	14.66	20.18	0.14	189.82
PSA density (μg)	0.026	0.016	0.429	0	4.23
BV (L)	2.96	2.81	3.11	2.06	4.19
PSA mass (μg)	1.34	0.81	2.23	0.03	11.15
Waist	87.2	82	92.6	56.2	132
Hip	97	93.8	101	69	164
WHR	0.90	0.86	0.93	0.60	1.24
SBP (mmHg)	126	115	138	77	237
DBP (mmHg)	80	73	88	41	155
Pulse	78	70	86	41	154
CHOL	5.04	4.44	5.68	1.88	18.91
TG	1.61	1.13	2.39	0.23	9.99
HDL	1.25	1.08	1.45	0.24	7.48
LDL	2.60	2.22	2.99	0.02	8.21
FBG	5.42	5.07	5.88	3.23	25.92
Alb	46.2	44.5	48	17.9	63.8
ALT	27	19	39	1.0	1111
AST	25	21	31	1.0	814
GGT	32	22	53	10	2676
ALP	87	75	103	21	677
TP	75.6	73	78.4	50.6	115.3
GLB	29.3	27.2	31.6	15.1	89.3
BUN	5.3	4.5	6.2	1.5	39.3
Cr	79.7	72.4	87.4	10.2	567.2
UA	385.8	337.4	441.7	102.8	1127.1

BMI, body mass index; PSA, prostate specific antigen; PV, prostate volume; BV, blood volume; WHR, waist-hip ratio; SBP, systolic pressure; DBP, diastolic blood pressure; CHOL, cholesterol; TG, triglyceride; HDL, high density lipoprotein; LDL, low density lipoprotein; FBG, fasting blood glucose; Alb, albumin; ALT, alanine aminotransferase; AST, aspartate aminotransferase; GGT, gamma-glutamyl transferase; ALP, alkaline phosphatase; TP, total protein; GLB, globulin; BUN, blood urea nitrogen; Cr, creatinine; UA, uric acid

### Correlation among BV, PV and PSA levels

The values of PSA, PSA density, and PSA mass were normally distributed after a log transformation. The univariable regression showed that PV, BV, systolic pressure (SBP), pulse, fasting blood glucose (FBG) and age were significantly associated with PSA level (*P* < 0.05) (Table 2). Furthermore, the multivariate regression analysis showed that PV, BV, FBG and age were significantly associated with PSA level (*P* < 0.05).

**Table 2:** Linear regression analysis for the associations between BV and PV with PSA level

Factors	Univariable regression			Multivariable regression		
	β	SE	*P* value	β	SE	*P* value
BV	− 0.118	0.005	< 0.01	− 0.109	0.005	< 0.01
PV	0.012	< 0.01	< 0.01	0.012	< 0.01	< 0.01
SBP	0.012	0.003	< 0.01	0.004	0.003	0.123
DBP	− 0.001	0.003	0.727			
Pulse	0.007	0.003	0.022	0.003	0.003	0.24
CHOL	− 0.005	0.003	0.075			
TG	− 0.003	0.002	0.149			
HDL	− 0.003	0.003	0.305			
LDL	0.002	0.002	0.138			
FBG	− 0.009	0.003	0.002	− 0.007	0.003	0.023
Alb	0.015	0.009	0.082			
ALT	0.001	0.003	0.640			
AST	− 0.001	0.005	0.863			
GGT	− 0.001	0.003	0.58			
ALP	0.003	0.003	0.434			
TP	− 0.009	0.006	0.156			
GLB	− 0.003	0.004	0.456			
BUN	0.004	0.007	0.535			
Cr	− 0.001	0.004	0.851			
UA	0.002	0.003	0.506			
Age	0.004	< 0.01	< 0.01	0.001	< 0.01	< 0.01

BMI, body mass index; PSA, prostate specific antigen; PV, prostate volume; BV, blood volume; WHR, waist-hip ratio; SBP, systolic pressure; DBP, diastolic blood pressure; CHOL, cholesterol; TG, triglyceride; HDL, high density lipoprotein; LDL, low density lipoprotein; FBG, fasting blood glucose; Alb, albumin; ALT, alanine aminotransferase; AST, aspartate aminotransferase; GGT, gamma-glutamyl transferase; ALP, alkaline phosphatase; TP, total protein; GLB, globulin; BUN, blood urea nitrogen; Cr, creatinine; UA, uric acid. SE, standard error

### Correlation among BMI and PSA-related parameters

Table 3 shows the relationship between WHR or BMI and PSA, PSA density and PSA mass in different WHR or BMI categories (normal weight, overweight and obese). FBG, age and WHR were used as independent variables, and the multivariate regression analysis showed that WHR was negatively associated with PSA and PSA density in all categories (Table 3). However, no significant association was found between WHR and PSA mass. The same results were found in the relationship between BMI and PSA, PSA density and PSA mass. BMI was negatively associated with PSA and PSA density in all categories, and no significant association between BMI and PSA mass was detected in all categories.

**Table 3:** Multivariate linear regressions for the associations of factors with PSA, PSA density and PSA mass

Factors	PSA			PSA density			PSA mass		
	β	SE	*P* value	β	SE	*P* value	β	SE	*P* value
WHR	− 0.149	0.02	< 0.01	−0.206	0.021	< 0.01	− 0.023	0.021	0.268
Normal weight	0.115	0.035	0.001	0.089	0.034	0.009	0.163	0.035	< 0.01
Overweight	− 0.157	0.034	< 0.01	− 0.175	0.033	< 0.01	− 0.129	0.034	< 0.01
Obese	−0.238	0.052	< 0.01	−0.272	0.052	< 0.01	−0.192	0.052	< 0.01
BMI	− 0.007	< 0.01	< 0.01	− 0.01	< 0.01	< 0.01	0	< 0.01	0.608
Normal weight	− 0.006	0.001	< 0.01	− 0.008	0.001	< 0.01	0.003	0.001	0.074
Overweight	− 0.008	0.002	< 0.01	− 0.011	0.002	< 0.01	− 0.001	0.002	0.406
Obese	−0.011	0.002	< 0.01	−0.013	0.002	< 0.01	−0.005	0.002	0.002

## Discussion

Our results demonstrated that PV was positively associated with serum PSA concentrations, while BMI and BV were inversely related with PSA levels, indicating that BMI, BV and PV should be taken into account when recommending a patient to take prostate biopsy based on serum PSA concentrations. Furthermore, the present study demonstrated that a higher BMI might be associated with a larger PV and BV. In addition, PSA-related parameters (PSA density and PSA mass) associated with different BMI categories were introduced in this study and it was demonstrated that PSA mass was not related to BMI in Chinese men.

Our results showed that serum PSA concentrations decreased with the increase in BMI among the participants who were not diagnosed with prostate cancer. This confirms the results from previous studies which have shown an inverse correlation between BMI and serum PSA [5–8, 12–14]. Obesity plays a key role in developing abnormalities in sex hormone metabolism and insulin levels, as a result of the excessive accumulation of adipose tissue or body fat. It can lead to the benign prostatic enlargement by raising the estrogen and estradiol levels, while lowering testosterone and serum globulin-binding protein levels [15]. The elevated estrogen/testosterone ratio associated with obesity might increase the stromal/epithelial cell ratio in benign prostatic hyperplasia nodules [16].

Previous studies have demonstrated that higher BMI might be associated with larger BV [8, 12], which could bias real serum PSA concentrations, and this finding was confirmed in the present study. The underlying hypothesis is that the amount of PSA released from cells in the prostate would be diluted to a lower concentration in men with larger BV in comparison with the one with smaller BV. Moreover, we found that the BMI was positively correlated with PV, whilst PV was positively correlated with the level of PSA. However, the BMI was negatively correlated with PSA level. One possible explanation is that, on one hand, higher BMI might cause larger PV and increase PSA levels, and on the other hand, higher BMI could cause hemodilution because the BV has increased, and the hemodilution of blood volume on PSA was more remarkable than the increase in PSA caused by PV [8, 17].

Due to the impact of BV and PV on PSA levels, it is necessary to make a comprehensive judgment by combining BV and PV. Some new PSA parameters have been proposed to eliminate the effects of these factors on PSA and improve the sensitivity and specificity of prostate cancer screening. We estimated PSA density and PSA mass, respectively, as PSA concentration divided by PV and PSA concentration times BV, and showed that PSA density concentration was inversely correlated with BMI, but PSA mass showed no significant correlation with BMI. Our analysis results indicated that assessment of PSA concentration by using PSA mass will not be affected by obesity in Chinese men. The previous study also showed that there was no association between BMI and PSA mass that relates to prostate cancer screening in our study. Furthermore, we also introduced WHR to analyze the relationship between obesity and PSA because PSA density and PSA mass were related to height and weight, and it might lead to the misjudgment of relationship between obesity and PSA. The obesity was represented by both WHR and BMI, and it was found that the results obtained from both WHR and BMI showed a consistent trend.

There were some limitations in our study. First, this is a cross-sectional study. Second, because the study participants were Chinese, the data may not necessarily represent populations outside China. We have analyzed the data according to WHO’s BMI standard grading system as it is the globally recognized standard to assess somebody’s weight. However, Asians have smaller skeletal frame and it is significant to perform further analysis according to the BMI standard for Asian adults and compare the results of these two systems. Moreover, the study measures did not include total levels of testosterone, which is an important indicator, as it was not routinely measured. The serum total testosterone is inversely associated with BMI, and obesity is usually directly associated with the low testosterone levels and causes many systemic illnesses. Neither did the present study perform analysis of other obesity indices except BMI. In addition, the socioeconomic factors and other potential confounders which might influence the BMI and PSA levels were not introduced into this study. However, our findings are consistent with those of numerous studies conducted in other regions and population.

## Conclusions

The results of this large-sample, hospital-based study in China indicate that PV is positively associated with PSA concentration, but BMI and BV are inversely correlated with PSA concentration. Otherwise, PSA mass might be the best parameter to estimate the PSA concentration without being affected by obesity in Chinese men.

## Data Availability

The data of the current study are available from the corresponding author on reasonable request.

## Abbreviations

BMI: Body mass index
PSA: Prostate-specific antigen
PV: Prostate volume
BV: Blood volume
SE: Standard error
WHR: Waist-hip ratio
SBP: Systolic pressure
DBP: Diastolic blood pressure
CHOL: Cholesterol
TG: Triglyceride
HDL: High density lipoprotein
LDL: Low density lipoprotein
FBG: Fasting blood glucose
Alb: Albumin
ALT: Alanine aminotransferase
AST: Aspartate aminotransferase
GGT: Gamma-glutamyltransferase
ALP: Alkaline phosphatase
TP: Total protein
GLB: Globulin
BUN: Blood urea nitrogen
Cr: Creatinine
UA: Uric acid

## References

[1] Ferlay J, Soerjomataram I, Dikshit R, Eser S, Mathers C, Rebelo M, Parkin DM, Forman D, Bray F (2015). Cancer incidence and mortality worldwide: sources, methods and major patterns in GLOBOCAN 2012. Int J Cancer.

[2] All cancers estimated incidence, mortality and prevalence of all cancers (excluding non-melanoma skin cancer) in 2012. J Natl Cancer Inst. 2017;109(9). https://doi.org/10.1093/jnci/djx205 10.1093/jnci/djx205 28954292

[3] Dunn MW (2017). Prostate cancer screening. Semin Oncol Nurs.

[4] Catalona WJ, Smith DS, Ratliff TL, Dodds KM, Coplen DE, Yuan JJ, Petros JA, Andriole GL (1991). Measurement of prostate-specific antigen in serum as a screening test for prostate cancer. New Engl J Med.

[5] Fowke JH, Matthews CE (2010). PSA and body composition by dual X-ray absorptiometry (DXA) in NHANES. Prostate.

[6] Loeb S, Carter HB, Schaeffer EM, Ferrucci L, Kettermann A, Metter EJ (2009). Should prostate specific antigen be adjusted for body mass index? Data from the Baltimore longitudinal study of aging. J Urol.

[7] Price MM, Hamilton RJ, Robertson CN, Butts MC, Freedland SJ (2008). Body mass index, prostate-specific antigen, and digital rectal examination findings among participants in a prostate cancer screening clinic. Urology.

[8] Banez LL, Hamilton RJ, Partin AW, Vollmer RT, Sun L, Rodriguez C, Wang Y, Terris MK, Aronson WJ, Presti JC, Kane CJ, Amling CL, Moul JW, Freedland SJ (2007). Obesity-related plasma hemodilution and PSA concentration among men with prostate cancer. JAMA.

[9] Martinez-Pineiro L, Garcia Mediero JM, Gonzalez Gancedo P, Tabernero A, Lozano D, Lopez-Tello JJ, Alonso-Dorrego JM, Nunez C, Picazo ML, Madero R, De La Pena JJ (2004). Probability of prostate cancer as a function of the percentage of free prostate-specific antigen in patients with a non-suspicious rectal examination and total prostate-specific antigen of 4–10 ng/ml. World J Urol.

[10] Yang HJ, Doo SW, Yang WJ, Song YS (2012). Which obesity index best correlates with prostate volume, prostate-specific antigen, and lower urinary tract symptoms?. Urology.

[11] Yelsel K, Alma E, Eken A, Gulum M, Ercil H, Ayyildiz A (2015). Effect of obesity on International Prostate Symptom Score and prostate volume. Urol Ann.

[12] Grubb RL, Black A, Izmirlian G, Hickey TP, Pinsky PF, Mabie JE, Riley TL, Ragard LR, Prorok PC, Berg CD, Crawford ED, Church TR, Andriole GL, Team PP (2009). Serum prostate-specific antigen hemodilution among obese men undergoing screening in the prostate, lung, colorectal, and ovarian cancer screening trial. Cancer Epidemiol Biomark Prev.

[13] Bonn SE, Sjolander A, Tillander A, Wiklund F, Gronberg H, Balter K (2016). Body mass index in relation to serum prostate-specific antigen levels and prostate cancer risk. Int J Cancer.

[14] Sarma AV, Jaffe CA, Schottenfeld D, Dunn R, Montie JE, Cooney KA, Wei JT (2002). Insulin-like growth factor-1, insulin-like growth factor binding protein-3, and body mass index: clinical correlates of prostate volume among Black men. Urology.

[15] Pasquali R, Casimirri F, Cantobelli S, Melchionda N, Morselli Labate AM, Fabbri R, Capelli M, Bortoluzzi L (1991). Effect of obesity and body fat distribution on sex hormones and insulin in men. Metabolism.

[16] Giovannucci E, Rimm EB, Chute CG, Kawachi I, Colditz GA, Stampfer MJ, Willett WC (1994). Obesity and benign prostatic hyperplasia. Am J Epidemiol.

[17] Walsh PC (2005). The association of body mass index and prostate-specific antigen in a population-based study. J Urol.

